# Evaluation of independent risk factors associated with surgical site infections from caesarean section

**DOI:** 10.1007/s00404-022-06885-7

**Published:** 2022-12-26

**Authors:** Matthew Erritty, Joann Hale, James Thomas, Anna Thompson, Ria Wright, Anna Low, Megan Carr, Richard George, Lisa Williams, Alexandra Dumitrescu, Jacqui Rees, Shashi Irukulla, Jonathan Robin, Christopher H. Fry, David Fluck, Thang S. Han

**Affiliations:** 1 Obstetrics and Gynaecology, Ashford and St Peter’s Hospitals NHS Foundation Trust, Guildford Road, Chertsey, KT16 0PZ Surrey UK; 2 Surgical Site Infection Surveillance Team, Ashford and St Peter’s Hospitals NHS Foundation Trust, Guildford Road, Chertsey, KT16 0PZ Surrey UK; 3 Department of Quality, Ashford and St Peter’s Hospitals NHS Foundation Trust, Guildford Road, Chertsey, KT16 0PZ Surrey UK; 4 Department Acute Medicine, Ashford and St Peter’s Hospitals NHS Foundation Trust, Guildford Road, Chertsey, KT16 0PZ Surrey UK; 5 School of Physiology, Pharmacology and Neuroscience, University of Bristol, Bristol, BS8 1TD UK; 6 Department of Cardiology, Ashford and St Peter’s Hospitals NHS Foundation Trust, Guildford Road, Chertsey, KT16 0PZ Surrey UK; 7 Department of Endocrinology, Ashford and St Peter’s Hospitals NHS Foundation Trust, Guildford Road, Chertsey, KT16 0PZ Surrey UK; 8 Institute of Cardiovascular Research, Royal Holloway, University of London, Egham, TW20 0EX Surrey UK

**Keywords:** BMI, Ethnicity, Smoking, Emergency C-section

## Abstract

**Background:**

The present study assessed factors associated with the risk of surgical site infections (SSI) after a caesarean section (C-section).

**Methods:**

Data were collected in 1682 women undergoing elective (53.9%) and emergency (46.1%) C-sections between 1st August 2020, and 30th December 2021, at a National Health Service hospital (Surrey, UK).

**Results:**

At the time of C-section, the mean age was 33.1 yr (SD ± 5.2). Compared to women with BMI < 30 kg/m^2^, those with a BMI ≥ 35 kg/m^2^ had a greater risk of SSI, OR 4.07 (95%CI 2.48–6.69). Women with a history of smoking had a greater risk of SSI than those who had never smoked, OR 1.69 (95%CI 1.05–2.27). Women with a BMI ≥ 30 kg/m^2^ and had a smoking history or emergency C-section had 3- to tenfold increases for these adverse outcomes. Ethnic minority, diabetes or previous C-section did not associate with any of the outcomes.

**Conclusions:**

High BMI, smoking, and emergency C-section are independent risk factors for SSI from C-section. Women planning conception should avoid excess body weight and smoking. Women with diabetes and from ethnic minority backgrounds did not have increased risks of SSI, indicating a consistent standard of care for all patients.

## What does this study add to the clinical work

**Table:** 

The presence of a high BMI amongst patients with a history of smoking, or those undergoing emergency C-section accentuated the risk of adverse outcomes to C-section. Our findings should serve as an important message to women of child-bearing age to avoid excess body weight and not to start smoking.	

## Introduction

Caesarean section (C-section) is one of the most common obstetric surgical procedures [1]. Surgical site infections (SSIs) have been reported in at least 3–18% of patients after undergoing a C-section [2–4], with the majority (84–89%) occurring in the community within one month of hospital discharge [2, 5].

A number of factors have variably been reported to associate with SSI, with conflicting findings, possibly due to a lack of control of confounding factors. In one study, subcutaneous haematoma after the procedure and a higher body mass index (BMI) at admission were associated with a greater risk [6], whilst antibiotic prophylaxis pre-incision [6, 7] or after the operation [6], and vaginal preparation with iodine-povidone solution and spontaneous placenta removal were associated with a lower risk of SSI [7]. Other studies identified further risk factors, including: chorioamnionitis and anaemia (or blood loss requiring transfusion), amniotomy or premature ruptured membrane more than six hours, and emergency C-section to be risk factors [8–10]. Demographic and socioeconomic status include age, low socioeconomic status or fewer years of education [8, 10] and tobacco use, whilst chronic conditions such prepregnant hypertension and gestational diabetes, and high parity have also been documented to associate with SSI [10]. On the other hand, the occurrence of SSI amongst ethnic minority groups, particularly in high income countries, has not been well-researched. In this study, using multivariable logistic regression analysis, we sought to determine independent risk factors for SSI, in women admitted consecutively for C-section delivery.

## Methods

### Study design, participants and setting

We carried out an analysis of prospectively collected data over seventeen months in women undergoing C-section delivery at a National Health Service hospital. This hospital serves a catchment area of about 430,000 people.

### Management and outcome measures

Patient characteristics including age, ethnicity, smoking status and medical history (type of diabetes and previous C- section), and BMI (weight in kg divided by square of height in m) was measured at booking (about 20 weeks of gestation). We followed the Getting It Right First Time (GIRFT) programme. This initiative aimed to improve outcomes of C-section by implementing an SSI prevention care bundle in maternity, in addition to the standard care, including: vaginal cleansing and negative pressure dressing [11]. Vaginal cleansing was with 4% chlorhexidine (*ChloraPrep*, Becton, Dickinson U.K. Limited) solution. The national guideline to reduce SSI in patients undergoing C-section recommends in all NHS hospitals the use of chlorhexidine as the first choice and povidone iodine (*PVP- I*, CAS 25655–41-8, Life Science, Performance Chemicals, UK) as an alternative if chlorhexidine is contra-indicated [12]. Negative pressure wound therapy dressings (*PICO*, Smith & Nephew plc, UK) were used. In accordance with NICE guideline, preoperative antimicrobial were offered to all patients [13]. Indications for postoperative antimicrobial therapy were based on a suspicion of infections, including chorioamnionitis, urinary tract infections, or sepsis; however a substantial proportion were given as a prophylaxis.

Absorbable synthetic monofilament suture (*Monocryl*^™^, Johnson & Johnson Medical Ltd.) was used for subcutaneous suture of the skin, and absorbable synthetic braided suture (*Vicryl*^™^, Ethicon, Inc.) for all intrabdominal sutures. Closure of the abdominal wall fat layer was encouraged when it exceeded 2 cm in depth with either *Monocryl* or *Vicryl*. SSI were defined as superficial incisional, deep incisional, or organ/space [14]. The rates of SSI after C-section were recorded using the Getting It Right First Time proforma [11] and entered onto the Maternity Badgernet database (routinely used by maternity services in the UK) [15]. For completeness of data collection, every patient was contacted directly and six weeks postnatally to capture SSIs that might have been treated by a general practitioner.

### Categorisation

For the purpose of analysis, categorisation of SSIs was created. This was based on patients who did not acquire an SSI (low-risk group) and those who had superficial incisional, deep incisional or organ/space SSIs (high-risk group). Age was grouped approximately by decades 18–29.9, 30–39.9, and ≥ 40 years; ethnicity into two categories: white British and all those from South Asians, Afro-Caribbeans, Africans, mixed race and other ethnicities; diabetes into two groups: no history of diabetes and any of gestational diabetes (diet or drug treatment) or type 1 and type 2 diabetes; smoking into two groups: never and a history of smoking (former or current smokers); BMI was categorised into three groups: < 30, 30–34.9, and ≥ 35 kg/m^2^, and pre-operative antimicrobial prophylaxis into two groups: no treatment and treatment with any type of antibiotics.

### Statistical analysis

Chi-squared tests were used to assess differences between SSI and risk factors (age, ethnicity, previous C-section, diabetes mellitus, smoking status, BMI, pre-operative antimicrobial prophylaxis, vaginal cleansing, and emergency C-section). Logistic regression was conducted using risk factors (independent variables) to predict SSI (dependent variables). Data are presented in two models; model 1: univariable (unadjusted) analysis, performed by entering each independent variable individually into regression equations; model 2: multivariable analysis, performed by entering all independent variables simultaneously into regression equations, *i.e.* variables were adjusted for one another to minimise confounding effects. Odds ratios (OR) are given with 95% confidence intervals (CI). Analyses were performed using IBM SPSS Statistics, v25.0 (IBM Corp., Armonk, NY).

## Results

From a total of 1691 women undergoing C-section between 1st August 2020 and 30th December 2021, data were available for 1682 (99.5%) women, 54.8% of whom underwent elective C-section, and 45.2% an emergency procedure. On average, 99 women underwent C-section a month, with monthly admissions being at a relatively constant level. The mean age was 33.1 years (SD = 5.2), and their age was normally distributed with the highest frequency occurring in the 30–34.9 age band. Most women were white British (72.8%), followed by South Asians (15.8%), Afro-Caribbeans or Africans (2.5%), mixed race (2.3%) and other ethnicities (3.2%), whilst that of the remaining 3.4% of women was unknown. There were 9.7% current and 6.1% former smokers. Gestational diabetes was identified in 13.9%, of whom 9.0% were treated with diet and lifestyle medication and 4.9% with an anti-hyperglycaemic agent. There were only 0.7% with a history of either type 1 or type 2 diabetes. Patients with BMI in the categories of 30–34.9 and ≥ 35 kg/m^2^ were 14.5% and 11.3%, respectively. The overall rates of pre-operative and post-operative anti-microbial prophylaxis were 81.8% and 23.8%, and the rate for either pre- or post-operative treatment was 86.6%, i.e. 13.4% did not have any treatment. Vaginal cleansing was applied to 45.1% of women. Superficial incisional SSI occurred in 6.4%, whilst deep incisional SSI only occurred in 0.5% of the sample population, and there were no organ/space SSI (Table 1).

**Table 1:** Characteristics of 1682 women undergoing Caesarean section

	Distribution	
	*n*	%
Age (years)		
18.3–24.9	115	6.8
25–29.9	324	19.3
30–34.9	627	37.3
35–39.9	478	28.4
40–54.6	138	8.2
Ethnicity		
Whites	1225	72.8
Asians	265	15.8
Blacks	42	2.5
Mixed race	39	2.3
Other ethnicities	53	3.2
Not recorded	58	3.4
Smoking status		
Non-smoker	1417	84.2
Current smoker	163	9.7
Former smoker	102	6.1
Body mass index (kg/m^2^)		
< 30	1248	74.2
30–34.9	244	14.5
35–39.9	122	7.3
≥ 40	68	4.0
Diabetes status		
No history of diabetes	1421	84.5
Gestational diabetes controlled by diet	167	9.9
Gestational diabetes controlled by drugs	83	4.9
Type 1 and type 2	11	0.7
Previous C-section	527	31.3
Emergency C-section	761	45.2
Vaginal cleansing with 4% chlorhexidine gluconate		
Yes	759	45.1
No	923	54.9
Pre-operative antimicrobial prophylaxis		
None	306	18.2
Ceftriaxone-metronidazole	1165	69.3
Cefuroxime	78	4.6
Clindamycin	32	1.9
Co-amoxiclav	38	2.3
Others	63	3.7
Post-operative antimicrobial prophylaxis		
None	1281	76.2
Yes	401	23.8
Surgical site infection		
None	1566	93.1
Superficial incisional	108	6.4
Deep incisional	8	0.5
Organ/space	0	0

**Fig. 1:**
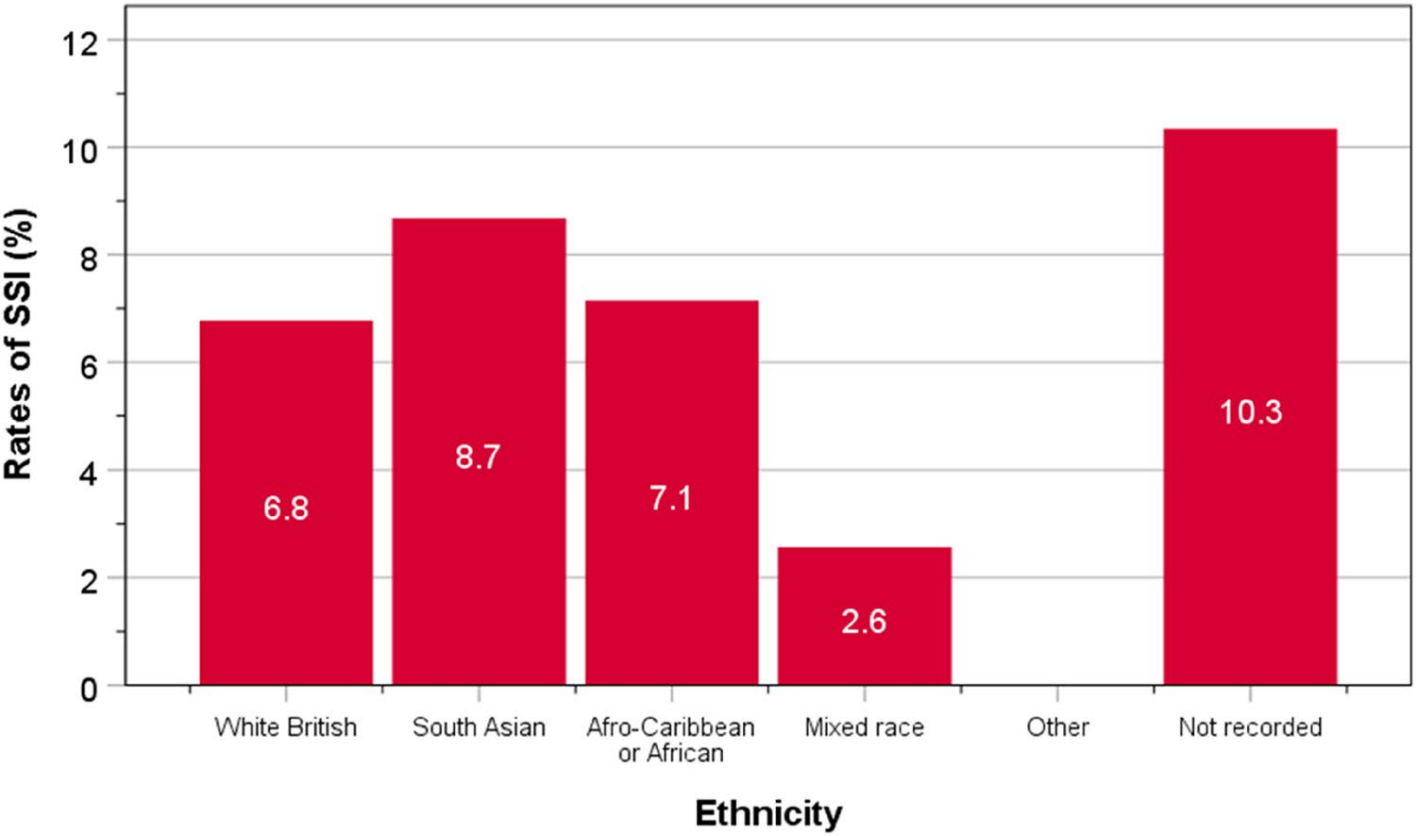
Rates of SSI in different ethnic groups

The rates of SSI after C-section amongst ethnic groups did not differ significantly (*P* = 0.573): 6.8% in white British, 8.7% in South Asians, 7.1% in Afro-Caribbeans, 2.6% in mixed race, and none in other ethnic groups (Fig. 1). Overall, the rates of SSI amongst white British and those amongst all ethnic minority groups were identical (6.8%, *P* = 0.573).

Amongst patients with BMI in the categories of < 30, 30–34.9, and ≥ 35 kg/m^2^, the rates of SSI rose from 0.9 to 2.5 and 3.2% (*P* < 0.001). SSI occurred in 6.2% in those who never smoked and rose to 10.6% in former or current smokers (*P* = 0.010).

The proportions of patients of different ages, ethnicity, BMI, history of previous C-section equally received either or both pre-operative and post-operative antimicrobial prophylaxis/treatment. Significantly higher proportions of patients with a history of smoking, diabetes or emergency C-section, and those who had vaginal cleansing received either or both pre-operative and post-operative antimicrobial prophylaxis/treatment (Table 2).

**Table 2:** Rates of preoperative antimicrobial prophylaxis given to different groups of patients

	Pre-operative antimicrobial prophylaxis			Post-operative antimicrobial prophylaxis/treatment		
	Rates (%)	*χ*^2^	*P*	Rates (%)	*χ*^2^	*P*
Age (years)						
18.3–29.9	82.8	0.1	0.975	87.7	1.2	0.543
30–39.9	81.8			86.0		
40–54.6	81.2			88.4		
Ethnicity						
White British	82.4	5.2	0.075	87.2	2.2	0.336
Ethic minority groups^a^	81.5			85.7		
Not recorded	70.7			81.0		
Smoking status						
Never-smoker	80.4	12.3	** < 0.001**	85.5	1.5	** < 0.001**
Former/current smokers	89.4			92.8		
BMI (kg/m^2^)						
< 30	82.7	3.4	0.186	87.0	1.1	0.569
30–34.9	80.7			86.5		
≥ 35	77.4			84.2		
Diabetes status						
No history of diabetes	80.9	4.7	**0.016**	85.6	7.8	**0.003**
Diabetes mellitus^b^	86.6			92.0		
Previous C-section						
No	81.3	0.6	0.233	86.7	0	0.938
Yes	82.9			86.5		
Admission type						
Elective C-section	79.0	10.4	**0.001**	85.3	2.9	0.052
Emergency C-section	85.2			88.2		
Vaginal cleansing						
Yes	86.3	22.3	** < 0.001**	90.8	24.8	** < 0.001**
No	77.3			82.4		

^a^Black, South Asians, mixed race, and other ethnicities.

^b^Gestational diabetes controlled by diet or drugs, and Type 1 or type 2 diabetes

There were no differences in SSI between ethnic groups, history of diabetes or previous C-section (Table 3). Multivariable logistic regression analysis showed that compared to women with a BMI < 30 kg/m^2^, those with a BMI of 30–34.9 kg/m^2^ had a greater risk of SSI: OR 2.39 (95%CI 1.46–3.91); and for those with a BMI ≥ 35 kg/m^2^, the risk was further accentuated: OR 4.07 (95%CI 2.48–6.69). Compared with women who never smoked, those with a history of smoking had greater risk of SSI: OR 1.69 (95%CI 1.05–2. 27) (Table 4).

**Table 3:** Proportions of patients with SSI according to different factors

	Surgical site infection		
	Rates	Group differences	
	%	*χ*^2^	*P*
Age band (years)			
18.3–29.9	7.7	1.1	0.571
30–39.9	6.4		
40–54.6	8.0		
Ethnicity			
White British	6.8	1.1	0.573
Ethic minority groups^a^	6.8		
Not recorded	10.3		
Smoking history			
Never smokers	6.2	6.6	**0.010**
Former/current smokers	10.6		
BMI (kg/m^2^)			
< 30	0.9	8.9	**0.003**
30–34.9	2.5		
≥ 35	3.2		
Diabetes status			
No history of diabetes	6.7	0.6	0.425
Diabetes mellitus^b^	8.0		
Previous C-section			
None	6.3	2.7	0.062
Yes	8.5		
Admission type			
Elective	6.5	0.5	0.279
Emergency C-section	7.4		
Pre-operative antimicrobial prophylaxis			
Yes	6.2	0.3	0.352
No	7.0		
Post-operative antimicrobial prophylaxis or treatment			
Yes	7.7	0.6	0.257
No	6.6		
Vaginal cleansing			
Yes	5.7	3.3	**0.043**
No	7.9		

^a^Afro-Caribbeans or Africans, South Asians, mixed race, and other ethnicities. ^b^Gestational diabetes controlled by diet or drugs, and Type 1 or type 2 diabetes

**Table 4:** Univariate and multivariable logistic regression analysis to assess the association between risk factors and outcomes

	Risk for SSI		
Univariable analysis	OR	95%CI	*P*
Age			
18.3–29.9	1.22	0.80–1.87	0.354
30–39.9 (reference)	1	–	–
40–54.6	1.26	0.65–2.44	0.491
BMI (kg/m^2^)			
< 30 (reference)	1	–	–
30–34.9	2.36	1.46–3.83	** < 0.001**
≥ 35	3.71	2.32–5.93	** < 0.001**
Smoking history			
Never smokers (reference)	1	–	–
Former/current smokers	1.78	1.14–2.79	**0.011**
Admission for C-section			
Elective (reference)	1	–	–
Emergency C-section	1.14	0.78–1.66	0.497
Vaginal cleansing			
Yes (reference)	1	–	–
No	1.43	0.97–2.11	0.072
Multivariable analysis^a^			
BMI (kg/m^2^)			
< 30 (reference)	1	–	–
30–34.9	2.39	1.46–3.91	** < 0.001**
≥ 35	4.07	2.48–6.69	** < 0.001**
Smoking history			
Never smokers (reference)	1	–	–
Former/current smokers	1.69	1.05–2. 27	**0.032**

^a^Multivariable model included BMI, smoking, elective/emergency C-section admission, age, ethnicity, diabetes, previous C-section, pre-operative antimicrobial prophylaxis and vaginal cleansing analysed simultaneously (only variables significantly associated with outcomes are presented)

**Fig. 2:**
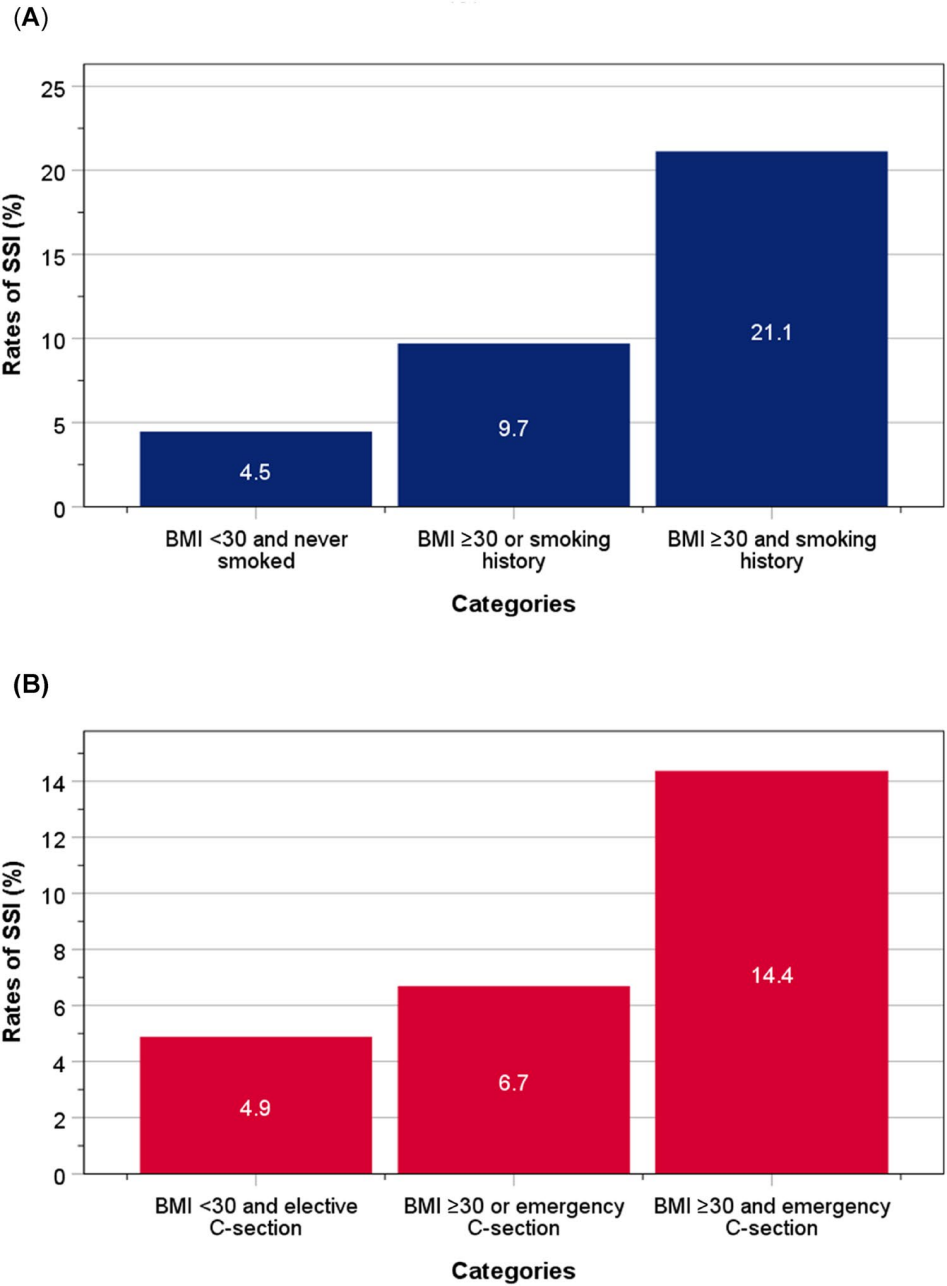
Rates of SSI according to BMI and smoking status (**A**), or BMI and emergency C-section (**B**)

Amongst categories of BMI < 30 kg/m^2^ and who never smoked, BMI ≥ 30 kg/m^2^ or a history of smoking, and BMI ≥ 30 kg/m^2^ and a history of smoking, the rates of SSI rose from 4.5 to 9.7, and 21.1% (*χ*^2^ = 38.9, *P* < 0.001) (Fig. 2A). Amongst categories of BMI < 30 kg/m^2^ and an elective C-section, BMI ≥ 30 kg/m^2^ or an emergency C-section, and BMI ≥ 30 kg/m^2^ and an emergency C-section, the rates of SSI rose from 5.1 to 6.7, and 14.4% (*χ*^2^ = 21.4, *P* < 0.001) (Fig. 2B).

Multivariable logistic regression, with adjustments for age, ethnicity, history of diabetes previous C-section, pre-operative antimicrobial prophylaxis and vaginal cleansing showed that compared to women with a BMI < 30 kg/m^2^ and who never smoked, those with a BMI ≥ 30 kg/m^2^ and a smoking history, the risk accentuated further for SSI: event rates = 4.5% vs 21.1%; OR 5.74 (95%CI 3.03–10.89). Similarly, compared to women with BMI < 30 kg/m^2^ and undergoing an elective C-section, those with a BMI ≥ 30 kg/m^2^ and who were undergoing emergency C-section had a further elevated risk of SSI: event rates = 4.9% vs 14.4%; OR 3.26 (95%CI 1.94–5.48).

## Discussion

This study found that for a C-section a high BMI at booking (≥ 35 kg/m^2^) and a smoking history were independent risk factors for SSIs. Furthermore, the risk was accentuated amongst smokers and those undergoing emergency C-section who had a high BMI. We also observed that women from ethnic minority groups, those with diabetes or a previous C-section did not have an increased risk of SSI arising from C-section delivery.

Of the significant independent risk factors observed in our study, a high BMI consistently emerged as the most powerful predictor of SSI after a C-section, and is consistent with findings from previous studies [6, 8–10]. Patients with a history of smoking (former and current smokers) were also at increased risk of SSI, despite higher proportions being covered with pre-operative antimicrobial prophylaxis. High BMI was also shown to be an added risk amongst patients undergoing an emergency C-section. Both BMI and smoking are modifiable risk factors, and the observation that the combination of these two factors accentuated the risk of adverse outcomes arising from a C-section suggest that women of pregnancy age should, if possible, avoid excess body weight and never start smoking. This could also serve a springboard for lifelong healthy habits to prevent cardiometabolic conditions, such as diabetes mellitus and cardiovascular disease [16].

The pathophysiological mechanisms for increased SSIs amongst smokers are thought to involve carbon monoxide, nitric oxide and nicotine. These agents may have a direct action on endothelial dysfunction or other vasoactive effects that may lead to postoperative tissues necrosis [17], impairment of the inflammatory healing response or bacteriocidal mechanisms and delay proliferative healing responses [18]. With respect to the increased risk of SSI amongst women undergoing emergency C-section, it is possible that they had less preparation time for adequate treatment, such as a full maternity care bundle, including timely provision of prophylactic antimicrobials. Such a group of patients may also have had other non-pregnancy-related acute conditions, such as a respiratory infection.

There has been a national drive to improve health inequalities, including patients from areas of high deprivation and those from ethnic minority communities [19]. There is a lack of research on the relationship between ethnicities and the incidence of SSIs after a C-section, particularly in high income countries. Our study showed that patients from ethnic minority groups, mostly Asians and Afro-Caribbeans or Africans, had similar levels of risk as white British for SSI. This provides evidence that health care inequalities amongst patients undergoing C-section from different ethnicities have been eliminated at our hospital.

Meta-analysis and systematic reviews on antibiotic prophylaxis and SSI showed conflicting findings due to heterogeneity of different combinations of agents [7, 20, 21]. However, based on available evidence, pre-operative antimicrobial prophylaxis is recommended by national guidelines for routine use in patients undergoing C-section [13]. The lack of association between the use of pre-operative antimicrobial prophylaxis in our cross-sectional study does not necessary indicate that this treatment is ineffective. It is likely that the choice to treat or not in this study appeared to be appropriate, since those with low risk did not need an unnecessary treatment. This is an important decision which helps reduce widespread use of antibiotics, and thus minimises the potential for antibiotic resistance. For example, proportionally more high-risk patients such as smokers or those with diabetes were covered with pre-operative antimicrobial prophylaxis. This decision helps explain the reason where patients with underlying diabetes in this study were not at increased risk of SSI, in contrast to a previous report [10]. Our observation of the benefit of vaginal cleansing is consistent with findings from previous studies [22, 23].

We also observed that patients with diabetes did not have greater risks of SSI compared with those without the condition. This is likely due to better approaches to clinical care of patients, including greater cover with antimicrobial prophylaxis (as observed in this study), as well as good glycaemic control provided to this high-risk group. A specialist centre (Stephanie Marks Diabetes Centre) at our hospital has been established for over a decade, employing an antenatal-endocrine team to provide joint care of every pregnant woman with diabetes [24].

Absorbable subcutaneous sutures were used for all patients at our hospital. Previous studies comparing the rates of SSI between absorbable subcutaneous sutures and non‐absorbable staples found inconsistent results. Some reported absorbable sutures were associated with lower risks of SSI [25], whilst other found no differences [26].

### Strengths and limitations

The strengths of this study include the large sample of consecutive admissions, and completeness of recruitment (99.5%). The study contains a wide range of principal risk factors and outcome measures, in addition to SSIs, including sepsis, reoperation, and readmission from SSI. The independent risk factors were robustly examined using multivariable analysis where all predictor variables were entered simultaneously into the regression equations to eliminate potential confounding effects between each other. This technique has often been overlooked in previous studies resulting in conflicting findings from previous reports. Our study is limited by its cross-sectional design, therefore certain findings such as post-operative antimicrobial treatment and outcomes, should be interpreted with caution. In a separate study of this cohort of patients, we have demonstrated that antimicrobial treatment was associated with significant reduction in SSI [27]. However, further prospective case–control studies would be necessary for assessing the effectiveness of treatment and outcomes.

## Conclusions

The presence of a high BMI amongst patients with a history of smoking, or those undergoing emergency C-section accentuated the risk of adverse outcomes to C-section. Our findings should serve as an important message to women of child-bearing age to avoid excess body weight and not to start smoking. Women with diabetes and from an ethnic minority background did not have increased risk of SSI, indicating a consistently good standard of care-equality.

## Data Availability

Not applicable.
